# Family-centered depression treatment for older men in primary care: a qualitative study of stakeholder perspectives

**DOI:** 10.1186/s12875-017-0659-4

**Published:** 2017-09-29

**Authors:** Ladson Hinton, Andrés F. Sciolla, Jürgen Unützer, Edward Elizarraras, Richard L. Kravitz, Ester Carolina Apesoa-Varano

**Affiliations:** 1 Department of Psychiatry and Behavioral Sciences, University of California Davis, 2230 Stockton Blvd., Sacramento, CA 95618 USA; 2 Department of Psychiatry, University of California Davis, 2230 Stockton Blvd., Sacramento, CA 95618 USA; 3 Department of Psychiatry and Behavioral Sciences, 1959 NE Pacific Street, Seattle, WA 98195 USA; 4 Department of Biology, San Francisco State University, 1600 Holloway Avenue, San Francisco, CA 94132 USA; 5 Division of General Internal Medicine, University of California Davis, 4150 V Street, Sacramento, CA 95817 USA; 6 Betty Irene School of Nursing, University of California Davis, 4610 X Street, Sacramento, CA 95817 USA

**Keywords:** Depression, Older men, Family, Qualitative

## Abstract

**Background:**

Family members often play important roles in the lives of depressed older men and frequently attend primary care visits with their loved ones, yet surprisingly little is known about how to most effectively engage and include family members in depression treatment. However, including family in depression treatment may be difficult due to several factors, such as depression stigma and family conflicts. The objective of this study was to describe challenges in engaging family members in older men’s depression treatment and potential strategies to overcome those challenges.

**Methods:**

A cross-sectional, qualitative descriptive interview study was conducted in a safety-net, Federally Qualified Health Center in California’s Central Valley. A total of 37 stakeholders were recruited, including 15 depressed older (i.e. age ≥ 60) men, 12 family members, and 10 clinic staff. Depressed men were identified through mail outreach, waiting room screening, and referral. Depressed men identified family members who were later approached to participate. We also recruited a purposeful sample of clinic staff. Interviews explored stakeholder perspectives on family involvement in men’s depression treatment as part of a primary care intervention. Interviews were conducted using a semi-structured interview guide, tape-recorded, transcribed verbatim, and translated if the interview was conducted in Spanish.

**Results:**

Four themes were identified representing core challenges: engaging men at the right time; preserving men’s sense of autonomy; managing privacy concerns; and navigating family tensions. Stakeholders also provided practical suggestions and advice about how each of these challenges might be addressed.

**Conclusions:**

While engaging family is a promising approach to strengthen depression care for older men in primary care settings, several potential challenges exist. Family- centered depression intervention development and clinical practice need to anticipate these challenges and to develop approaches and guidelines to address them.

## Background

Advancing depression care for depressed older men is a public health priority because older men are less likely than older women to receive depression treatment and are also more likely to commit suicide [1]. While prior research has demonstrated the challenges in getting depressed men to seek help for depression [2], relatively little is known about how to tailor treatments to more effectively engage and treat depressed men [3]. Mobilizing family members, who are often already assisting older adults in their healthcare, to support depression treatment has been suggested as one possible strategy that may help to improve outcomes and more effectively engage under-served groups such as older men and minorities [4–6]. Despite the possible value of including family members as a strategy to close gaps in care for depressed older men, little is known about the most effective ways to do this in primary care settings.

The potential value of family involvement in supporting older men’s depression treatment is supported by both observational studies and intervention studies. Men often rely on family members, particularly their wives and daughters, for assistance with managing their health and health care, including medication management, health-care decision-making, transportation to doctor’s appointments, and participating in healthcare visits [7–11]. More than one third of older adults who present for care in primary care settings are accompanied by family members, usually a spouse or adult child [12, 13]. Families often play a very influential role in getting depressed or suicidal men into formal treatment [1, 14–16]. Primary care providers view family members as potential allies in depression treatment [5] and a majority of older men prefer to have family involved in their care [6]. While we are not aware of any depression intervention studies that have systematically incorporated family members into treatment, one review of the literature found that family involvement in psychosocial interventions for chronic illness can improve outcomes [17].

Family members may also hinder depression care [4]. Family communication patterns that are critical, controlling or overprotective are associated with negative health and mental health outcomes [18, 19]. When family members view depression as a highly-stigmatized condition, a normal response to loss or aging, or as a part of co-occurring medical conditions, they may discourage help-seeking or be critical of the older man with depression [20–22]. Families may also view depression as “unmanly” and encourage men to “tough it out” rather than seek help [23]. Family attitudes towards antidepressants and endorsement of depression-related stigma can negatively influence patient adherence [24, 25].

While the literature highlights the potential value and challenges of engaging family to support older men’s depression care, little is known about how to address these challenges. In this manuscript, we describe the results of formative work (i.e. qualitative interviews with key stakeholders) as part of a larger effort to develop and test a family-centered intervention for ethnically diverse older men with depression in primary care settings. Specifically, we describe stakeholder perspectives on both the challenges in engaging family members in men’s depression treatment and the potential strategies to address those challenges. The larger goal of the project was to develop and test a primary-care based intervention to strengthen evidence-based depression treatments (e.g. behavioral activation, anti-depressant medications) for older men through the involvement of family members.

## Methods

### Setting

Participants were recruited from two primary care clinics at San Joaquin General Hospital (SJGH), a large county hospital in California’s Central Valley that provides care for underserved populations including a high proportion of Mexican Americans.

### Participant selection and recruitment

Three different stakeholder groups were recruited: depressed older men, their family members, and clinic staff, including those with administrative responsibilities.

#### Recruitment of depressed older men

Fifteen men were recruited from the San Joaquin General Hospital primary care clinics. The study inclusion criteria were as follows: (1) age 60 or older; (2) English or Spanish speaking; (3) non-demented; (4) non-psychotic; and (5) have a PHQ-9 score ≥ 10 [26]; and (6) expressed a willingness to have a family member be interviewed as part of the study. Three different recruitment approaches were used. Letters were mailed to 115 men with a chart diagnosis of depression or a PHQ-9 score ≥ 10 in the past year, 15 of whom returned the postcard expressing interest in participating and 10 were successfully interviewed. We also screened 123 men age 60 and above at the time of their primary care appointments and 23 had a score of ≥10. Of those 23, 4 were interviewed (13 did not have a family member, 5 declined, and one was lost to follow-up). Finally, 7 patients were referred directly from primary care physicians (PCPs) and 1 was successfully interviewed (2 declined, 2 were ineligible, and 2 were lost to follow-up).

#### Recruitment of family members

Of the 15 depressed men who were interviewed, 12 identified family members who were then successfully recruited and interviewed. For the remaining 3 men, their family members either could not be reached or declined to be interviewed.

#### Recruitment of clinic staff

We conducted a purposive sampling to identify 10 staff including administrators, primary care physicians, social workers and physician assistants. Because insights regarding the topic of engaging family in men’s depression care might vary depending on staff role and training, we chose to interview a variety of different types of staff to generate a wider range of perspectives.

### In-depth interviews

A qualitative approach was chosen to allow us to elicit and better understand stakeholder attitudes and concerns related to engaging family in older men’s depression care, a topic about which very little is known. In-depth interviews with stakeholders were conducted by interviewers who were bilingual/bicultural and who received training and supervision in conducting qualitative interviews. The interviews were semi-structured and included pre-determined topics and associated probes. These pre-determined topics were chosen by the research team to elicit in an open-ended fashion stakeholder perspectives on involving family in men’s depression treatment and to explore stakeholders’ perspectives on specific aspects of the research team’s anticipated intervention approach. For older men and family members, the topics included family relationships and living situation, depression explanatory model and views of treatment, attitudes and experiences related to family involvement in depression care, and best approaches to involve family in older men’s depression care. For clinic staff, the topics included background and role in clinic, attitudes and experiences in having family involved in depression treatment, and best approaches to involve family in older men’s depression treatment. The interviewer had latitude to explore topics and emerging themes as they arose during the interview.

Interviews were conducted separately with depressed older men and their identified family members. Interviews were conducted in the interviewee’s home and in their language of choice, either English or Spanish, and lasted 0.5 to 2.0 h. We interviewed clinic staff in their offices or by phone. Clinic staff interviews lasted 0.5–1.5 h. All interviews were digitally audio-recorded, transcribed verbatim into text by bilingual research staff and de-identified. Interviews conducted in Spanish were transcribed into English by bilingual research staff.

### Data analysis

Computer-assisted data analysis (i.e. data management and coding) was conducted using NVIVO® 10.0 (QSR International) and involved multiple steps that included open-coding and constant comparison, following an accepted approach for qualitative descriptive studies [27–29]. A descriptive approach was chosen because the goal of the study was to generate knowledge that would directly and pragmatically inform intervention development and implementation [30, 31]. First, three members of the research team, including two who were bilingual in Spanish and English, independently read and coded the interviews to identify emergent themes (i.e. meaning units of text) that related to family involvement in men’s depression care. The entire research team, which included a sociologist and two clinicians, then met to discuss their coding and through a consensus process grouped conceptually related themes. This process led to the identification of four themes representing core challenges related to family involvement in men’s depression treatment. A code book was developed and used by two members of the research team to systematically code the data for each of these core challenges. In this second round of coding we also identified mentions of possible strategies relevant to address each challenge in intervention development and clinical practice. Data saturation was reached for each of the four core challenges described in this study [32].

## Results

### Participant characteristics

Older men had a mean age of 63.5 years, were ethnically diverse (47% Latino) and 8 (53%) were married. Most family caregivers were either spouses (50%, *n* = 6) or adult children (25%, *n* = 3) and 42% (*n* = 5) were Latino (see Table 1 for additional characteristics). Clinic staff included physicians, social workers, nurses, physician assistants and clinic administrators (see Table 2 for additional characteristics).

**Table 1:** Characteristics of interviewed depressed older men and their family members

	Men (*N* = 15)	Family (*N* = 12)
Mean age	63.5	53.7
Ethnicity		
Latino	7 (46.66%)	5 (41.67%)
White non-Hispanic	4 (26.67%)	5 (41.67%
Other	4 (26.67%)	2 (16.66%)
Language		
English	12 (80%)	11 (91.67%)
Spanish	3 (20%)	1 (8.33%)
Country of birth		
United States	11 (73.34%)	11 (91.67%)
Mexico	2 (13.33%)	1 (8.33%)
Other	2 (13.33%)	0
Self-reported general health		
Excellent/very good	0	4 (33.34%)
Good	5 (33.33%)	5 (41.67%)
Fair	6 (40%)	2 (16.66%)
Poor	4 (26.67%)	1 (8.33%)
Relationship		
Spouse/partner	N/A	6 (50%)
Child	N/A	3 (25%)
Other family relative	N/A	2 (16.67%)
Family friend	N/A	1 (8.33%)
Marital status		
Married	8 (53.33%)	6 (50%)
Living in a marriage-like relationship	0	1 (8.33%)
Separated/divorced/widowed	4 (26.67%)	3 (25%)
Never married	3 (20%)	2 (16.67%)
Education		
< 12 years	4 (26.67%)	4 (33.33%)
12 years	5 (33.33%)	4 (33.33%)
> 12 years	6 (40%)	4 (33.33%)
Employment		
No, retired	9 (60%)	5 (41.67%)
No, unemployed but seeking work	5 (33.33%)	2 (16.67%)
Yes, part-time	0	1 (8.33%)
Yes, full-time	1 (6.67%)	4 (33.33%)
Income		
Less than $10,000	7 (46.67%)	3 (25%)
$10,000–$25,000	7 (46.67%)	5 (41.67%)
$25,000–$50,000	1 (6.66%)	4 (33.33%)

**Table 2:** Interviewed clinic staff characteristics

Characteristics	N (%)
Age	
20–29 years	2 (20%)
30–39 years	1 (10%)
40–49 years	4 (40%)
50–59 years	3 (30%)
60+ years	0
Gender	
Female	5 (50%)
Male	5 (50%)
Ethnicity	
Latino	5 (50%)
White non-Hispanic	5 (50%)
Role in clinic	
Non-MD clinical staff	5 (50%)
Physicians	4 (40%)
Administrators	1 (10%)
Years in practice	
1–10 Years	5 (50%)
11–20 Years	3 (30%)
> 20 Years	2 (20%)

### Qualitative findings

Analysis of the interviews with key stakeholders (i.e., depressed men, family members of depressed older men, and clinic staff) led to the identification of four themes related to core challenges in the implementation of a family-centered intervention for depressed older men in primary care: engaging men at the right time, preserving men’s sense of autonomy, managing privacy concerns, and navigating family tensions. In their discussions of these themes, stakeholders also made practical suggestions about how these challenges might be overcome.

### *“They got to be ready”*: engaging men at the right time

Stakeholders emphasized that the right time to engage men in a family-centered treatment is after they recognize that they are depressed and accepted the need for treatment. The wife of a depressed man told us that it would be difficult to engage her husband because “*I guess he thinks he doesn’t have a problem*.” Minimizing depressive symptoms or attributing them to other causes were cited as reasons men might not self-identify as depressed. Stakeholders emphasized that depression might need to be severe (e.g., “*they have to be willing, they have to hit rock bottom*”) before men are willing to accept any treatment: “*I think the person has to have a desire to do something. You know, it’s like that saying I heard before, too. It’s I’m sick and tired of being sick and tired” (depressed older man).* Stigma may also play an important role in men’s reluctance to recognize their depression and seek help: “*Well you have to get past the stigma of depression first…” (social worker).* Once they have accepted the diagnosis of depression, it may also take men time to warm up to the idea of family involvement. As one older man told us: *“But I wouldn’t force that [family involvement] because I do kind of believe is when you are ready and the reason why is for example like I would’ve rebelled if I didn’t, if you tried to force me with people.”*

Stakeholders suggested that for the reasons cited above, some men are unlikely to accept family involvement at the beginning of their depression treatment. As one primary care physician told us: “*I have to kind of work with them and initially gain their trust to even introduce the possibility of depression.”* Another clinic staff emphasized the need for a more gradual approach that begins by helping men gain insight before engaging family members: “*I think we need to work with a patient first to help them gain a better insight to their situation, to move them from, say pre-contemplative to contemplative stage, so that they’re more willing to hear their family and accept their support that they would have from their family.”* This was echoed by a depressed older man who said: *“First the person gets to know what’s going on with me and then they you know”, “would you mind if I ask your wife or somebody from your family to participate in the next visit?”* Thus, for a subgroup of men, it might make sense to broach the topic of family involvement after several sessions or even in the middle stages of treatment.

### *“They have to know they’re in charge”*: preserving men’s sense of autonomy

Stakeholders also stressed that involving family members might be off-putting or threatening to some men who may perceive it as undermining their sense of autonomy and control. The value placed on autonomy by men was viewed as closely connected to men’s masculine self-identity, as illustrated in the following quote from a clinician: “*You’re telling the patient, ‘I don’t think you can handle this on your own, I want to bring someone else to help you handle it’. And this is a gentleman who’s been living for sixty years plus, some or most of it on his own, you know.” Even if he’s had a family, he’s been at the head of the family, he’s been at the top of his game, now you’re telling him, “you’re going to need some help”.* Some participants viewed older men as particularly vulnerable to real or perceived threats to their autonomy because they often experienced other losses that undermined their independence and sense of usefulness, such as physical disabilities or job loss. A wife graphically described the importance of independence for her depressed husband: “*Sometimes a man is hard to help cause they, they want to do it or, you know, they don’t want to have you do it for them. And so that’s one thing I kind of have to keep holding myself back a lot of times when he’s eating.* […] *I want to help him out, but uh, I realized that him being a man he wants to try, he wants to do it himself.*” Another potential obstacle is that men might perceive family members as too controlling: “…*and the bottom line is this man feels as though his family member attempts to control what he does and that it’s under the guise of I’m trying to help you.”*

While emphasizing men’s need for control as a challenge, stakeholders also suggested that a family-centered intervention could be effective if it valued and addressed men’s need for control while negotiating a role for family involvement. As a clinic provider told us: “*I think that [family involvement] can be helpful as long as that doesn’t take the control and autonomy away from the patient.”* Along the same lines, a family member stressed that for the model to work, *“…the person who has depression has to know that they have that they’re in charge.”* One depressed older man emphasized that allowing men to feel that they are “in charge” might begin by respecting their preferences in who to involve in their care and for the clinician not to make assumptions about which family member to involve*.* Clinic staff suggested other possible strategies for helping men feel in charge of their treatment, such as managing their own medications: “*try to work out a system that the older man is in charge of their own medication, so that you empower them to be in charge*.” Asked if family might assist men in identifying side effects and remembering to take their medication, another clinician said, “*Yeah I think that that can be helpful as long as that doesn’t take the control and autonomy away from the patient to, you don’t want to feel like they’re belittled.”*

### “*Some stuff is personal*”: managing privacy concerns

A variety of concerns were expressed about how the presence of a family member might interfere with the older men to discuss sensitive or “personal” issues with their healthcare provider. As one man said**: “** *I don’t know if, um, I have such mixed emotions, but I don’t know if we should have the family member involved in their meeting. I just don’t think that’s productive myself, and I’m going to explain to you why: because then the person might not open up to things, that is very private.”* This man went on to give an example of something he wouldn’t want to share with his family: “*I had kept secret that I was almost killed working for the FBI in (the) US Marshalls office, and never told them to what degree, that I could’ve been killed, and I never shared to them that I had suffered emotionally from that*.” Additional reasons for not wanting to share things with family members included not wanting to “burden” them or feeling uncomfortable about showing emotions. As one man said “*Well, because no one wants to hear about your little problems when they got problems of their own*.” Stakeholders provided other topics that men might not feel comfortable discussing with family in the room, such as drug or alcohol use or sexual dysfunction. One provider echoed these concerns about the need for privacy around mental health issues: *“Yes, so the one thing is that there’s somebody else that they trust to that level that they are going to allow [them] to be in that circle of privacy, of knowing their mental or health issues. That’s number one: is there’s somebody there they can trust? Sometimes even their very close partners spouse may not be the right person for them to trust, because there might be some issues.”* Finally, some providers expressed concerns about sharing information with a family member from a medical-legal perspective (e.g., signing a HIPPA form or verbal consent). While some insisted patients would be required to sign a “release of information form”, others indicated that it was sufficient for the patient to give verbal consent when a family member was present.

To address the concerns about privacy in the context of a family-centered intervention, stakeholders said it would be important to give men the opportunity to spend at least some time talking individually with their provider while being treated for depression. Preserving this “one-on-one” time was viewed as important because as one depressed older man told us, “… *some of the things that I talked about was personal.”* This might also give providers a chance to query men about topics they would prefer not to discuss in the presence of family members.

### *“When the family is angry”*: navigating family tensions

Strained family relationships may make it difficult to engage and work effectively with family members in treatment. Sharing his reluctance to involve family members, one provider said: *“There might be a lot of family issues and problems because of like maybe even a family you know is almost you know there’s maybe there’s a lot of arguments and a lot of issues.”* Older men discussed how strained relationships might make family involvement difficult. Referring to his wife, one man said: “*It scares me sometimes because we’ll get on it about something like that right there, and she turns everything around that it’s my fault.”* Speaking about the possibility of having his brother participate in his depression care another man said: “*Yeah because like for me, me and my brother it would be a bad thing for him to be around where I can look at him and it’d be it’d be just a big fight. All I do is close my mind down*.” Some marital relationship may be so frayed that the partner is not willing to help, as described by a social worker: “*So what levels of you know ‘Screw you I, I don’t really care about you anymore, I’ve stopped loving you a long time ago. Now you want my help?’ You know so um there’s that like what level of investment does the family have in helping this person who they may have all this antipathy for?”*

While highlighting friction and tensions that might exist between men and family members, stakeholders also pointed to approaches that might help to overcome these challenges. One older man suggested “ground rules” to facilitate more constructive involvement of family: *“That would be everybody on an even ground. We already have it pre-limited to where there would be no yelling, no cussing, ground rules.”* An important part of getting people on “even ground” is to establish a common understanding of what depression is and the nature of the treatment model. One of the depressed older men said: “*Maybe the family is angry at the person who is suffering and so there’s issues there that need to be addressed, education-wise. And there is some tender and loving care that needs to be done there, from that perspective.*” A family member emphasized that education might be empowering to family members because “*if you know more about depression you know how to handle it*.” This man and several others highlighted the need to “educate” family members, either about the nature of depression, its treatment or about how the family might be more supportive to help men recover from their depression. As a social worker told us, *“I wonder if family members had some basic motivational interviewing techniques, could they use that to support the family member maybe in moving a little closer to education, moving a little closer to behavioral activation and other treatment interventions. So sometimes their approach is a bit abrasive and it’s purely in my opinion lack of education.”* Thus, stakeholders voiced their opinion that a successful intervention would require anticipating and managing potential or emerging family conflict, as well as educating family members.

## Discussion

There is a growing consensus that our health care system should be both person and family-centered to address the pressing needs of older adults who suffer from chronic health problems, including depression and other mental health conditions [33]. This study adds to our understanding of the types of challenges to involving family in depression care and how these challenges might be addressed in the development and implementation of a family-centered treatment model for depression. Through in-depth qualitative interviews with those who have the most at stake in a family-centered intervention model – depressed older men, family members, and providers - we identified four themes that represent core challenges as well as strategies to address them that are relevant for for both clinical reseach and primary care practice.

The core themes identified in this study are consistent with a growing literature on men, mental health and masculinity [34]. Men may be reluctant to seek help for depression or to accept the diagnosis because depression carries a “double-stigma” [1], threatening both men’s masculine self-identity and their sense of normality (i.e. stigma of “craziness” or severe mental illness). The value men place on self-sufficiency and control, for example, reflect traditional maculine norms, such as stoicism, that may inhibit both the expression of distress and formal help-seeking [3]. In addition, the importance of privacy for older men may reflect concerns about appearing “weak” or “vulnerable” in the presence of family members, situations that may evoke discomfort and even shame. Loss of control and feelings of shame are central to older men’s experience of depression [35]. An important caveat is that there is considerable variability among men in their masculine self-image (i.e., multiple masculinities) potentially creating more openness among some men to both depression treatment and family involvement [36]. Finally, it is important to stress that men may value independence in some aspects of depression self-management (e.g. taking medications) which may also serve to affirm their masculinity and to preserve their autonomy.

Our findings are also consistent with prior studies highlighting the complexity of work with families in the context of both health and mental health problems [37]. Prior work has shown how family members may serve as both barriers and facilitators of depression care in primary care [4] and among Latinos in mental health treatment [38]. There is also a literature on families and depression highlighting how families may offer support to people who are depressed but can also be unsupportive or even trigger or worsen depression [39, 40]. It is worth noting that for older men their family helpers are most likely to be women (i.e. either wives or daughter/daughter-in-laws) and that these gender and generational differences may also influence the qualities and dynamics of provision of support. Together, these prior findings and ours strike a cautionary note about family involvement in depression care that underscores the need for better guidelines and models to inform clinical practice and intervention work. These guidelines might also be helpful more broadly for mental health professionals, many of whom have not received formal training on how and when to involve family members in depression treatments for older adults.

In their description of themes, stakeholders offered practical suggestions that may help guide clinicians and interventionists in overcoming potential challenges. In Table 3 we summarize the possible approaches that could be used by clinicians, drawing on the suggestions of stakeholders and extrapolating from these based on our own insights from our work in this area. In terms of the timing of approaching men about family involvement in their depression care, our study suggests that there is not a “one size fits all” approach. While for some men the right time may be at the start of treatment for others it may be the middle phase of treatment to allow more time to come to terms with their diagnosis and to build trust with their PCP. To preserve men’s sense of autonomy and control, our findings highlight the importance of having discussions with men to assess their values and preferences, both in terms of whether to involve family but also how family is to be involved and whether there are any “off-limits” topics. These discussions might also include discussion about any concerns related to losing control over the treatment process, not having “private time” with the provider, escalation of family tensions or conflicts, and burdening family with emotional troubles. Jointly educating older men and their families about the nature and treatment of depression, laying ground-rules, agreeing on an agenda for each session, and focusing on concrete behavioral tasks may help diffuse existing family tensions. Finally, providers might benefit from education about obtaining authorization from the patient to include family members in treatment. Finally, clinicians should also consider and assess the impact of participation in older men’s depression care on family members themselves; it is possible, for example, that in some cases older men may want to include family but this might not be in the best interests of the family member’s health or well-being.

**Table 3:** Summary of core challenges to engaging family in men’s depression treatment and possible strategies to overcome them

Challenge	Possible strategies
Engaging men at the right time	• Work with men to help them accept the diagnosis and need for treatment before involving family • Giving men time to “warm-up” to the idea of family involvement
Preserving men’s sense of autonomy	• Communicate centrality of men’s preferences and values in treatment • Assess men’s preferences for family role in specific aspects of treatment
Managing privacy concerns	• Educate clinicians about guidelines for involving family members (e.g. HIPAA guidelines) [41] • Allow men to specify topics that are “off-limits” in discussions with family • Allow flexibility for care manager to meet individually with patient • Address men’s concerns about burdening family members
Navigating family tensions	• Set ground rules for joint sessions • Educate family about the nature of depression • Focus on specific behavioral tasks that can be shared with family • Develop a jointly agreed upon agenda for each treatment session

This study has several limitations. First, caution should be used in drawing any conclusions about the gender-specific nature of our findings. Our study was focused on depressed older men and we lack a comparison group of older women and their family members that would allow us to examine gender similarities and differences. In addition, our interviews with staff did not include queries about gender differences. Second, while a strength of our study is the inclusion of multiple stakeholder perspectives to provide a richer description of these themes, systematic comparison of the viewpoints of the three stakeholder groups is beyond scope of this study. In addition, our study was not designed to systematically compare the perspectives of Latinos and white non-Hispanics (or other ethnic/racial groups) but rather to identify themes that were broadly reflected in the sample and were relevant to development and implementation of a common intervention. Finally, our sample is unique in certain ways (i.e. safety net clinic, men age 60–70, high representation of Latinos) and caution should be used in generalizing to other populations.

## Conclusion

As our healthcare system moves toward more patient- and family-centered care, we need to understand how family members can most effectively be engaged to support care for depression and other chronic conditions in primary care and other treatment settings. To our knowledge, this is the first study to identify these four core challenges to family-centered depression treatment delivered in primary are settings and to provide practical suggestions on how these challenges might be addressed. This study, including the challenges we experienced in recruiting participants, demonstrates that engaging family in the delivery of mental health services is not always straightforward or easy and requires careful assessment of the preferences, concerns, and needs of patients and family members. To advance the field, we now need to apply knowledge from this and other observational studies to develop and test family-enhanced depression interventions in real-world settings.
